# Vertical distribution of radionuclides in soil at the Semipalatinsk Test Site beyond its test locations

**DOI:** 10.1371/journal.pone.0278581

**Published:** 2023-01-06

**Authors:** Andrey Panitskiy, Yelena Syssoeva, Symbat Baigazy, Assiya Kunduzbayeva, Laura Kenzhina, Yelena Polivkina, Natalya Larionova, Pavel Krivitskiy, Almira Aidarkhanova

**Affiliations:** Institute of Radiation Safety and Ecology, NNC RK, Kurchatov, Kazakhstan; Universiti Teknologi Malaysia, MALAYSIA

## Abstract

Data on the vertical distribution of radionuclides in the soil is necessary to fully understand the radioecological situation around ecosystems, give predictive estimates to how safe crop products are and justify a rehabilitation strategy for radioactively contaminated areas. A study was conducted to investigate the vertical distribution of radionuclides in soils of the former Semipalatinsk Test Site (STS) territory beyond its testing sites, that is, in areas in which no nuclear weapons or nuclear effects of radiological warfare agents were tested. Soil was sampled layerwise all over the Semipalatinsk Test Site down to 30 cm deep at a 5-cm spacing. Most of high activity concentrations of radionuclides all over the study area were detected in the 0–5 cm soil layer. Activity concentrations of the major man-made radionuclides were determined in soil samples collected by γ-, β and α-spectrometry. As a result, ranges of activity concentrations of ^137^Cs, ^241^Am, ^90^Sr and ^239+240^Pu were determined in 0–5, 5–10, 10–15, 15–20, 20–25, 25–30 cm soil layers. In the conventionally ‘background’ area, the 0–5 cm soil layer, on average, contains (the percentage of total activity concentration across the soil profile depth): ^137^Cs– 83%, ^239+240^Pu– 87% and ^90^Sr– 38%. For the 1953 plume, these values were 92%, 83% and 73%, respectively. Values for the 1951 plume in the 0–5 cm soil layer were: ^137^Cs– 93%, ^239+240^Pu– 93% and ^90^Sr– 59%. The minimum concentration of radionuclides are observed 20–30 cm deep in all areas studied. ^90^Sr is the most mobile radionuclide from the perspective of its ability to travel deep down the soil. The study found out that the nuclide vertical migration rates downward in soils based on detected activity were as follows (in descending order): ^90^Sr– ^137^Cs– ^239+240^Pu– ^241^Am. Coefficients that determine the ratio of the activity concentration of the radionuclide in the 0–20 and 0–30 cm soil cover layers to that of this radionuclide in the 0–5 cm topsoil were calculated. These coefficients enable to estimate the radionuclide inventory at each soil sampling point from their activity concentration in the 0–5 cm soil layer.

## Introduction

**Fig 1:**
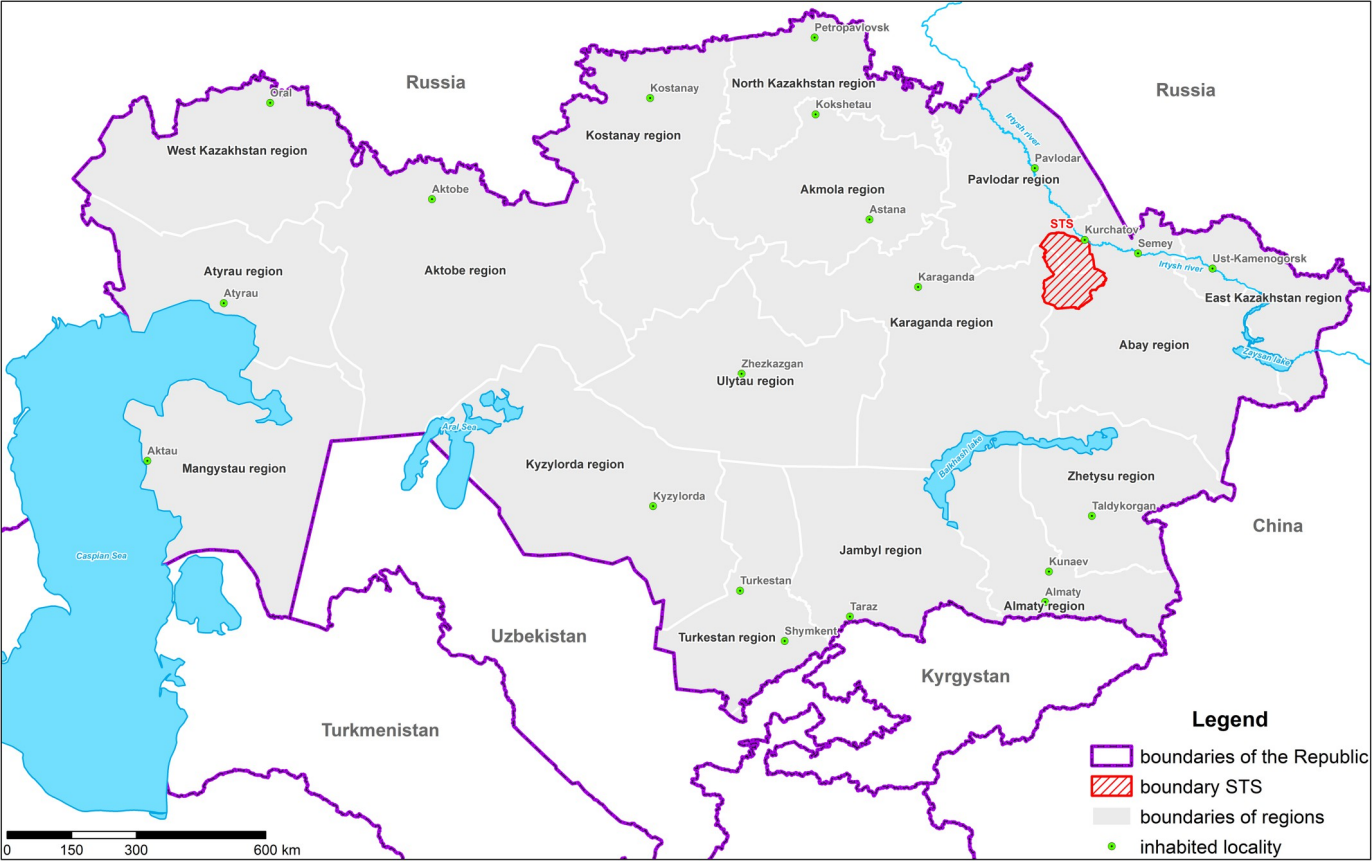
Schematic location of the STS territory in three regions of the Republic of Kazakhstan.

The former Semipalatinsk Test Site (STS) is located within three regions of the Republic of Kazakhstan–Karaganda, Pavlodar and Abai regions (the former Semey region) (Fig 1).

STS is characterized by having smaller testing sites at which different types of nuclear and fusion tests were conducted and damage effects of radiological warfare agents were tested. Between 1949 and 1989, 456 nuclear tests were conducted in the STS territory [1]. Those tests caused all environmental components to be contaminated both directly in test areas and beyond them (fallout in the form of plumes) [2–8]. 2008 through 2021, a comprehensive radioecological survey began in the Semipalatinsk Test Site territory, which was aimed at assessing whether it was possible to release a portion of the test site territory to the economic use. All environmental components–soil, plants, air, surface and ground waters, were surveyed. Under the survey, a territory, in which no nuclear weapon tests were directly conducted, was studied. That is, the area both within the Semipalatinsk Test Site and beyond its testing sites. For a comprehensive assessment of the radiological state of these areas, the vertical distribution of radionuclides in soil cover was researched. Results are of interest scientifically since they allow the vertical distribution nature of radionuclides in the soil to be addressed years after nuclear testing. Besides, data on the vertical distribution of radionuclides in soil is indispensable for decision-making on rehabilitation strategies for contaminated areas. Many studies have investigated the anthropogenic distribution in vertical soil profiles. In particular, the migration of radionuclides in contaminated soils as a result of radiation accidents at nuclear fuel cycle facilities (Radiation accident at the Southern Urals in 1957 and in Pripyat city in 1986 (Chernobyl NPP) has been well studied [9, 10]. Most of data was obtained for recent depositions shortly after accidents whereas, following testing at individual STS areas, more than 70 years have passed. A number of authors quote research findings on the relationship between features of the vertical migration of radionuclides in soil and its physical and chemical properties or the source species of radionuclides in soils [11–13] as well as depending on landscape conditions and on whether any external natural factors existed or not [14, 15]. In addition, soil and climatic conditions in areas of radiation accidents and at STS are significantly different.

Certain researchers quote data on the distribution of radionuclides in soils of forest ecosystems within the territory adjacent to STS [16]. Testing sites at STS, with respect to the vertical distribution of radionuclides have been also adequately studied whereas in areas beyond testing sites such research is sporadic [17–19].

This work aims at obtaining data on the vertical distribution of radionuclides in the soil cover of STS areas beyond testing sites, i.e. in areas in which no nuclear weapons or damage effects of radiological warfare agents were tested in order to form a general idea of the migration of radionuclides in vertical profiles of STS soils.

## Materials and methods

### Field activities

**Fig 2:**
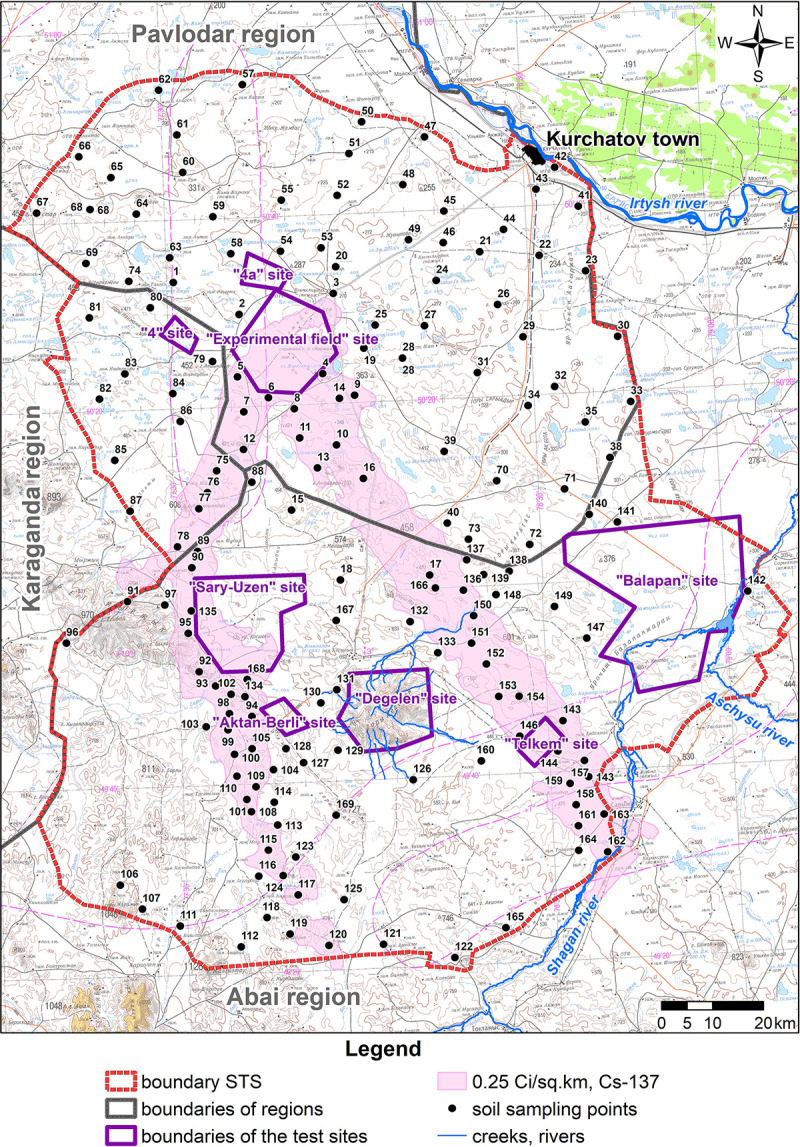
Schematic site preparation for layer-by-layer soil sampling within STS with fallout overlaid in the form of plumes (along the 0.25 Ci/km^2^ isoline for ^137^Cs).

In terms of soil and geography, the test site area covers two subzones of the steppe zone. Those are the subzone of desert steppes on light-chestnut soils and the subzone of dry steppes with a zonal subtype of chestnut soils. Light-chestnut soils occupy the central, eastern and southern STS parts, and chestnut soils are common in the west and northwest of the test site territory. The soil cover inside subzones is nonuniform, which is attributed to the composition of parent rocks, moisture conditions and the land form. Geni of underdeveloped, normal and solonetzic soils became the most common in the study area amidst zonal chestnut and light-chestnut soil subtypes [20]. To obtain data on the vertical distribution of radionuclides in the STS territory, 169 stratified sampling points were located to be analyzed for radionuclides. Soil layers were selected at 0–5, 5–10, 10–15, 15–20, 20–25, 25–30 cm intervals. Deeper than 30 cm, soil was not sampled because, previously, authors had not registered any numerical values of radionuclides in the soil of study areas deeper than 30 cm [18]. Points of layer-by-layer sampling were plotted onto the topographic map of the STS territory with isolines of fallout plumes (density of contamination with ^137^Cs) resulted from aboveground nuclear testing on September 24, 1951 and August 12, 1953 (Fig 2). Plumes mentioned were identified by aerial gamma-spectrometric survey in 1990 and 1991.

Altogether, 1014 stratified soil samples were collected. Points were selected based on one point per 100 square kilometers. Initially, soil was sampled in STS areas that are in Pavlodar and Karaganda regions over a relatively uniform grid. With such approach, registration of quantitative values of activity concentrations of radionuclides in soil samples was not always successful because it was below the detection limit of the methodological instrumentation in use. Therefore, for a large amount of figures, layer-by-layer soil sampling points in the Abai region were mainly located in areas of fallout plumes from nuclear tests (Fig 2). Each sample was collected with a trowel of preset geometry, 10×10×5 cm (length-width-height). The sampling area of each soil layer was 200 cm^2^ (2 trowels).

### Laboratory research

#### Radionuclide analysis

Soil samples collected were sieved through a 1 mm mesh and air dried (to constant weight) prior to the measurement. Activity concentrations of ^137^Cs and ^241^Am in samples of dry soil were determined with a γ-spectrometer Canberra GX-2020 in a plastic container in the form of a straight cylinder of 94 mm diameter. The height of a counting sample varied with mass, 300 to 500 g. The measurement time was at least 60 min. The minimum detectable activity (MDA) for ^137^Cs and ^241^Am isotopes were 2.0×10^−1^ and 2.4×10^−1^ Bq/kg. For the calibration of the γ-spectrometer, calibration sources IAEA-RGK-1 Potassium Sulfate, IaEa-RGTh-1 Torium Ore, IAEA-RGU-1 Uranium Ore diluted were used. Measurements were performed as per the measurement procedure with a gamma-spectrometer [21].

The activity concentration of ^90^Sr was determined with a β-spectrometer ‘Progres-BG’ (Russia) [22]. To do so, a 15 g subsample was collected from each soil sample by squaring following the γ-spectrometric analysis. ^90^Sr concentration was determined by direct measurement with a beta-spectrometer ‘Progres-BG’ on the aluminum mold of 70 mm diameter and 4 mm high. The height of a counting sample varied with density. The exposure time was 20 min. MDA was 100 Bq/kg. The determination accuracy of ^90^Sr activity concentration was verified by periodic measurements of the ^22^Na calibration reference source.

In cases where ^90^Sr activity concentration in the test sample was below MDA, it was determined using a radiochemical analysis. ^239+240^Pu activity concentration was also determined by a radiochemical method.

For the determination of activity concentrations of ^90^Sr and ^239+240^Pu isotopes, soil samples were measured by spectrometry preceded by the radiochemical decomposition [23]. ^90^Sr and ^239+240^Pu isotopes were extracted and isolated in 4 main stages: 1 –washing radionuclides out of the soil matrix by a complete chemical decomposition; 2 –removal of interfering radionuclides; 3 –preparation of counting samples and 4 –determination of activity concentrations of radionuclides.

The activity concentration of ^239+240^Pu in counting samples was determined by means of a α-spectrometer ‘Alpha-Analyst’ (‘CANBERRA’, USA) equipped with solid-state PIPS detectors. MDA of ^239+240^Pu was 1.2×10^−1^ Bq/kg. The α-spectrometer was calibrated using ^239^Pu calibration source manufactured by The Source Inc. (USA, Santa Fe) with certificate of calibration Ref. PO# 100060.

^90^Sr was determined by adding an yttrium carrier. ^90^Y activity concentration was determined with a liquid scintillation β-spectrometer ‘TRI CARB 3100 TR’. The chemical yields of yttrium and strontium (following ^90^Y precipitation) were determined by atomic emission spectrometry (ICP-AS). ^90^Sr activity concentration was determined from that of ^90^Y. MDA of ^90^Sr was 1.2×10^−1^ Bq/kg. For ICP-AS calibration, multi-element standard solutions of reference standards (RS) were used. Compositions of metals are manufactured by Inorganic Ventures IV-ICP-MS-71А having the nominal certified value of metal content equal to 10 mg/l with the 0.5% uncertainty of the certified value (dilution coefficient, k = 2).

#### Mapping

Maps quoted in the paper were constructed by means of ArcGIS software using digitized maps of the Republic of Kazakhstan that were acquired by the branch ‘Institute of Radiation Safety and Ecology’ NNC RK from the Republican Public State Enterprise the ‘National Mapping and Geodesic Fund’ of the Committee for Geodesy and Mapping. The Ministry of Digital Development, Innovations and Aerospace Industry of the Republic of Kazakhstan under the State Procurement Agreement No. 02-19/122 dated 2020-04-28.

#### Quality control

Research was conducted by means of the analytical and testing equipment that had passed the calibration test according to the Law of the Republic of Kazakhstan dated July 7, 2000 No. 53-II ‘On Ensuring Uniformity of Measurements’.

During the radiochemical analysis, the measurement quality was overseen. For that end, one ‘repeated’ sample intended for quality control and repeatability of analytical results was added to each batch of soil analytes. To control a hypothetical cross-contamination of samples, a ‘blank’ sample was also added to the batch. A ‘repeated’ test sample was randomly selected from a set of samples in the batch. A ‘blank’ sample was prepared from soil with a known content of radionuclides to be analyzed at the ‘background’ level. A ‘repeated’ test and a ‘blank’ sample were analyzed simultaneously with all the other samples.

### Processing of results

Prior to the processing of findings, 3 retrievals were made by activity concentrations of radionuclides. The first retrieval–values of activity concentrations of radionuclides in the soil from sampling points located in the conventionally ‘background’ territory, that is area that are not within STS testing sites or fallout plumes. The second retrieval–values of activity concentrations of radionuclides in the soil from sampling points located in the plume following the 1951 test. The third retrieval–values of activity concentrations of radionuclides in the soil from sampling points located in the plume from the 1953 test.

To provide information on levels of activity concentrations of radionuclides in soil layers, the arithmetic mean and the error were calculated for each soil layer of interest. Minimum and maximum radionuclide activity concentrations were also determined in each soil layer. The activity concentrations of radionuclides, which were lower than MDA, were taken as quantitative values in the calculations. That was attributed to the conservative approach of obtaining results. Prior to data processing, a critical analysis of activity concentration values of radionuclides was carried out so as to identify sampling points with a possible anthropogenic impact or the impact by burrowers on the distribution nature of radionuclides in soil cover.

To determine the percentage of radionuclides in each soil layer, the total activity concentration was calculated across the soil profile depth by summing values of activity concentrations in each soil layer. Based upon data on the total activity concentration at each stratified soil sampling point, the abundance ratio of radionuclides in each soil layer was determined and expressed in percent of the total activity concentration at a sampling point. If detected, such points were excluded from the calculation. Next, values obtained for the abundance ratio of radionuclides were checked for any outliers of each sample to exclude artifacts. Data was verified as per the criterion equal to the standardized deviation of an outlier of interest [24]:

$$T=\frac{V-M}{\sigma }T_{st}$$ (1)

T–an outlier criterion;

V–artifacts;

М–the arithmetic mean of a test retrieval;

σ–the mean square deviation for a test retrieval;

Т_st_−standard values of the outlier criterion.

Based upon verification results, artefacts were excluded from the sample. The Shapiro-Wilk test was also applied to verify the hypothesis for the normality of data distribution in research.

## Results and discussion

Ranges of activity concentrations of radionuclides and their arithmetic mean in soil layers down to 30 cm at points of stratified soil sampling are listed in the table (Table 1). Results are listed for conventionally background areas and 1951 and 1953 plumes separately.

**Table 1:** Activity concentrations of radionuclides in soil layers down to 30 cm deep.

**Conventionally ‘background’ areas**									
**Depth, cm**	**Activity concentration, Bq/kg**								
	^**241**^**Am** (^a^n = 95)			^**137**^**Cs** (n = 75)		^**239+240**^**Pu** (n = 44)		^**90**^**Sr** (n = 86)	
	^**b**^ **AM±** ^**c**^ **SD**		**min-max**	**AM±SD**	**min-max**	**AM±SD**	**min-max**	**AM±SD**	**min-max**
0–5	4.9±1.2		0.4–81	27.8±3.3	5.4–193	121.7± 43.8	1–1600	7.2±0.73	1–42.0
5–10	0.9±0.1		0.2–7.7	2.7±0.5	0.2–20	8.0±4.0	0.1–160	5.6±0.68	0.1–29.0
10–15	0.7±0.06		0.1–3	0.7±0.06	0.2–3.8	1.7±0.7	0.1–25	3.4±0.39	0.1–18.3
15–20	0.6±0.06		0.2–3	0.5±0.04	0.2–2.2	1.6±0.8	0.1–30.3	2.9±0.41	0.1–19.4
20–25	0.6±0.04		0.1–3	0.6±0.04	0.1–2	0.9±0.5	0.1–13.7	3.7±0.57	0.1–17.6
25–30	0.6±0.05		0.1–2.5	0.5±0.04	0.1–2	3.1±2.3	0.1–69	3.2±0.57	0.1–16.5
**1953 plume**									
**Depth, cm**	**Activity concentration, Bq/kg**								
	^**241**^**Am** (n = 30)			^**137**^**Cs** (n = 9)		^**239+240**^**Pu** (n = 10)		^**90**^**Sr** (n = 9)	
	**AM±SD**	**min-max**		**AM±SD**	**min-max**	**AM±SD**	**min-max**	**AM±SD**	**min-max**
0–5	9.9±3.5	0.5–80		189.3±58.7	57.7–610	236.5±81.8	8.3–810	7.2±0.73	1–42.0
5–10	1.2±0.3	0.4–5.2		14.3±6.3	2.5–63	14.0±5.1	0.2–41.5	5.6±0.68	0.1–29.0
10–15	0.7±0.08	0.1–2.4		1.1±0.4	0.3–4.4	1.9±0.7	0.3–8	3.4±0.4	0.1–18.3
15–20	0.6±0.04	0.2–1.3		0.6±0.2	0.3–1.8	2.0±0.9	0.1–9.1	2.9±0.4	0.1–19.4
20–25	0.7±0.07	0.4–2.1		0.4±0.10	0.1–1.2	2.9±2.6	0.1–15.6	3.7±0.6	0.1–17.6
25–30	0.5±0.04	0.1–1.1		0.6±0.2	0.3–1.8	1.9±1.4	0.2–8.8	3.2±0.6	0.1–16.5
**1951 plume**									
**Depth, cm**	**Activity concentration, Bq/kg**								
	^**241**^**Am** (n = 19)			^**137**^**Cs** (n = 16)		^**239+240**^**Pu** (n = 6)		^**90**^**Sr** (n = 12)	
	**AM±SD**	**min-max**		**AM±SD**	**min-max**	**AM±SD**	**min-max**	**AM±SD**	**min-max**
0–5	19.5±5.0	2.7–75		141.8±17.9	33–240	130.5±30.5	42–260	27.8±7.4	6.3–91
5–10	1.1±0.2	0.3–3.5		7.4±1.5	1.5–21	8.9±3.18	0.9–19	7.0±1.2	2–13.5
10–15	0.9±0.4	0.2–7.8		1.3±0.3	0.1–4.5	1.0±0.4	0.1–2.7	5.7±1.97	0.3–25
15–20	0.6±0.05	0.2–1.2		0.5±0.07	0.1–0.8	0.5±0.2	0.1–1	1.8±0.4	0.4–5.7
20–25	0.6±0.07	0.1–1.5		0.6±0.1	0.1–2.4	0.2±0.05	0.1–0.4	0.6±0.06	0.2–0.9
25–30	0.5±0.05	0.1–1		0.5±0.06	0.1–0.9	0.1±0.01	0.1–0.13	0.2±0.02	0.1–0.3

^a^n–the number of soil sampling points with figures on activity concentrations of radionuclides in soil layers

^b^AM: arithmetic mean

^c^SD: standard deviation

Based upon findings, differences in ranges of activity concentration values in the 0–5 cm soil layer in the conventionally ‘background’ STS territory were found to be from 1 (for ^137^Cs and ^90^Sr) to 3 (for ^239+240^Pu) orders of magnitude. For the 1951 plume, differences in ranges of activity concentration values in the 0–5 cm soil layer vary from 1 (for ^137^Cs) to 3 (for ^90^Sr, ^241^Am and ^239+240^Pu) orders of magnitude. For the 1951 plume, differences in ranges of activity concentration values in the 0–5 cm soil layer for all radionuclides in question are 1 order of magnitude. Most of elevated values of radionuclide activity concentrations are noted at soil sampling points located in fallout plumes (Fig 1). In addition, maxima of radionuclide activity concentrations are observed in the soil layer down to 5 cm deep. Elevated values of radionuclide activity concentrations in the 0–5 cm soil layer were also registered for individual soil sampling points located in the conventionally ‘background’ STS territory. Local fallout plumes from other tests could have had an effect here.

To determine the percentage of radionuclidies in each soil layer, the total activity concentration across the soil profile was calculated by summing values of activity concentrations of radionuclides in all soil layers within each soil sampling point. Based upon data on the total activity concentration, the abundance ratio of radionuclides was determined in each soil layer expressed as the percentage of the total activity concentration of radionuclides at a sampling point. Values calculated for the abundance ratio of radionuclides were verified for the distribution normality. Shapiro-Wilk test values (with the significance point, р = 0.05) proved to be greater than theoretical ones for all retrievals, which allows the acceptance of a hypothesis for the distribution normality. The table (Table 2) lists values of the abundance ratio of radionuclides in each soil layer. In most cases, values of ^241^Am do not exceed MDA or quantitative values of its activity concentration are only observed in the 0–5 and 5–10 cm topsoil. Thus, consideration of the distribution pattern of this radionuclide in the vertical soil profile with this methodological approach does not seem possible. Therefore, data on the distribution of ^241^Am is not quoted from now on.

**Table 2:** Concentration ratios of radionuclides in the soil.

**Conventionally ‘background’ areas**						
**Depth, cm**	**% of the total activity concentration**					
	^**137**^**Cs** (n = 75)		^**239+240**^**Pu** (n = 44)		^**90**^**Sr** (n = 86)	
	**AM**	**min-max**	AM	**min-max**	AM	**min-max**
0–5	83.4	70.6–93.2	86.8	64.5–98.6	38.5	2.1–94.2
5–10	6.8	1.1–18.0	4.2	0.3–9.9	22.2	1.4–54.1
10–15	2.8	0.3–5.9	2.7	0.2–7.7	14.0	0.7–39.6
15–20	2.3	0.4–5.9	2.8	0.01–9.5	10.9	0.8–28.4
20–25	2.4	0.1–6.5	1.7	0.02–6.4	7.8	0.5–28.2
25–30	2.2	0.1–5.9	1.9	0.04–7.8	6.5	0.7–24.2
**1953 plume**						
**Depth, cm**	**% of the total activity concentration**					
	^**137**^**Cs** (n = 9)		^**239+240**^**Pu** (n = 10)		^**90**^**Sr** (n = 9)	
	**AM**	**min-max**	AM	**min-max**	AM	**min-max**
0–5	91.8	86.5–96.1	90.9	83.3–95.7	73.3	53.3–93.5
5–10	6.5	2.5–12.0	5.1	1.7–10.9	14.6	3.1–29.7
10–15	0.5	0.2–0.9	1.7	0.3–4.0	6.6	1.9–12.3
15–20	0.4	0.1–0.8	1.1	0.02–2.5	4.0	0.8–9.3
20–25	0.4	0.01–0.7	0.7	0.01–3.2	1.09	0.1–4.0
25–30	0.5	0.04–1.1	0.5	0.07–2.1	0.5	0.1–1.6
**1951 plume**						
**Depth, cm**	**% of the total activity concentration**					
	^**137**^**Cs** (n = 16)		^**239+240**^**Pu** (n = 6)		^**90**^**Sr** (n = 12)	
	**AM**	**min-max**	AM	**min-max**	AM	**min-max**
0–5	92.6	85.3–98.4	93.3	88.6–97.0	58.7	36.4–91.6
5–10	5.2	0.7–12.4	5.5	2.1–10.0	19.6	2.0–33.7
10–15	0.9	0.04–2.1	0.6	0.1–1.1	13.4	0.6–33.8
15–20	0.4	0.1–0.9	0.4	0.1–0.7	5.6	0.8–12.8
20–25	0.5	0.1–1.6	0.2	0.1–0.3	1.9	0.4–4.8
25–30	0.4	0.07–0.9	0.1	0.1–0.2	0.7	0.2–1.9

Maxima of activity concentrations of radionuclides all over the study area noted in the 0–5 cm soil layer. In the conventionally ‘background’ area, the 0–5 cm soil layer, on average, contains: ^137^Cs– 83%, ^239+240^Pu– 87% and ^90^Sr– 38%. For the 1953 plume, these values are 92%, 83% and 73%, respectively. Values for the 1951 plume in the 0–5 cm soil layer are: ^137^Cs– 93%, ^239+240^Pu– 93% and ^90^Sr– 59%. The minimum concentrations of radionuclides are observed at a depth of 20–30 cm in all areas studied.

Findings showed that despite the fact that over 60 years have passed since the end of aboveground tests, which contributed most to soil contamination with man-made radionuclides, the vertical migration of radionuclides in soil is minor. Slight penetration of radionuclides may be related to the fact that moistening can be the major factor affecting the migration of radionuclides deep down soil. The STS territory is under arid conditions, therefore, the topsoil wetting depth due to precipitation does not exceed 7–10 cm (subject to no additional moistening). Deeper than 10 cm, moisture is no longer the major factor that affects the travel of radionuclides deep down soil.

^137^Cs isotope in loamy soils is bound with the lattice of clay minerals in a non-exchangeable way. Therefore, the migration of this radionuclide is related to the movement of clay minerals. Chestnut soils dominate in the STS territory, the top layer in which (about 10 cm) is friable due to a large amount of fragmentary material of different size. Therefore, soil particles are spilled by the gravity force. Denser soil layers occur beneath this relatively friable one. Probably, that is why ^137^Cs, ^241^Am and ^239+240^Pu movement to these layers is hampered. This occurs because, for example, ^137^Cs travel with colloid mineral particles, and the behavior of Pu isotope is mostly defined by hydrolysis, which may occur with sufficient moisture. Moisture under STS conditions is only in topsoil (with no additional moisture).

An increased migration of ^90^Sr to lower soil layers is attributable to its soluble forms dominating in the soil of the STS territory [2]. It is particularly noticeable in conventionally ‘background’ areas (Table 2). More intense ^90^Sr vertical movement in conventionally ‘background’ areas relative to plumes from the 1951 and 1953 tests is attributable to the difference in its mobility rate in study areas. Previous research into the species of radionuclides showed that near fallout plumes from the aboveground tests mentioned contain less ^90^Sr exchangeable form compared to the adjacent conventionally ‘background’ areas [2, 25]. The difference in the content of ^90^Sr exchangeable form varies from 2 times to 2 orders of magnitude, that of the acid-soluble form–up to 2 orders of magnitude. This is attributed to the difference in conditions under which radioactive contamination is produced in these areas. The global fallout that mainly affected conventionally ‘background ‘areas is characterized by a higher mobility of artificial radionuclides than the ones incorporated in radioactive particles resulted from aboveground tests. A similar tendency towards an elevated penetration of radionuclides deep down the soil in conventionally ‘background’ areas is also noted for ^137^Cs and ^239+240^Pu.

As part of the comprehensive survey of the STS territory conducted 2008 through 2021, over 20,000 soil samples were collected at 0–5 cm deep. As a result, a large dataset was obtained characterizing the areal distribution of radionuclides in the STS topsoil. However, this data is oftentimes insufficient to estimate different radiation risks. For instance, the estimate of a possible content of radionuclides in crop products uses transfer factors of radionuclides from soil to plants, typically derived for soil at a depth of 0–20 cm [26, 27]. Data obtained during our research allows adequately reliable coefficients to be derived for each radionuclide to determine the ratio of a particular radionuclide in the soil cover layer of 0–20 cm to a particular radionuclide in the 0–5 cm topsoil (*K*_*n*,*0–20*_). In turn, this coefficient allows the conversion of activity concentrations of radionuclides from a depth of 0–5 cm, determined from the areal distribution of radionuclides 0–20 cm deep being necessary for further assessment of a possible content of radionuclides in crop products at each point of the test site with values of activity concentrations of radionuclides for the 0–5 cm soil layer.

Authors also found out previously that no artificial radionuclide are detectable in the soil deeper than 30 cm [17] in the test site area if there is no additional moistening in the form of creeks. Hence, one might assume that the radionuclide inventory in these areas is concentrated in the 0–30 cm soil layer. Thus, the data obtained allows us to determine a coefficient for the radionuclide activity ratio in the soil cover layer of 0–30 cm to the one in the 0–5 cm topsoil (*K*_*n*,*0–30*_). This enables to estimate the inventory of radionuclides at each soil sampling point from their activity concentrations in the 0–5 cm soil layer.

Values of *K*_*n*,*0-X*_ were computed as per the following formula:

$$K_{n,0-X}=\frac{A_{m,i,0-X}}{A_{m,i,0-5}}$$ (2)

where:

*K*_*n*,*0-X*_*−*the ratio of a radionuclide activity in the soil cover layer of 0–20 cm (or 0–30 cm one) to the one in 0–5 cm topsoil;

*A*_*m*,*i*,*0–5*_ *–*the activity concentration of a radionuclide in the 0–5 cm soil layer, Bq/kg;

*A*_*m*,*i*,*0-X*_*−*the activity concentration of a radionuclide in the 0–20 cm soil layer (or 0–30 cm one), Bq/kg.

Mean values and ranges of coefficients (*K*_*n*,*0–20*_ and *K*_*n*,*0–30*_), which determine the ratio of radionuclide activity concentrations in the soil cover layer down to 20 and 30 cm deep to the activity concentration of a radionuclide in the 0–5 cm topsoil, are listed in the table (Table 3).

**Table 3:** Values of coefficients (*K*_*n*,*0–20*_ and *K*_*n*,*0–30*_), which determine the ratio of radionuclide activity concentrations in the soil cover layer down to 20 and 30 cm deep to the activity concentration of a radionuclide in the 0–5 cm topsoil.

**Conventionally ‘background’ area**						
	^**137**^**Cs** (n = 75)		^**239+240**^**Pu** (n = 44)		^**90**^**Sr** (n = 86)	
	**AM**	**min-max**	**AM**	**min-max**	**AM**	**min-max**
***K***_***n*,*0–20***_	1,15	1,05–1,36	1,12	1,01–1,34	4,13	1,05–32,6
***K***_***n*,*0–30***_	1,20	1,07–1,42	1,17	1,01–1,55	5,50	1,06–48,7
**1953 plume**						
	^**137**^**Cs** (n = 9)		^**239+240**^**Pu** (n = 10)		^**90**^**Sr** (n = 9)	
	**AM**	**min-max**	**AM**	**min-max**	**AM**	**min-max**
***K***_***n*,*0–20***_	1,08	1,03–1,2	1,09	1,04–1,17	1,40	1,07–1,83
***K***_***n*,*0–30***_	1,09	1,04–1,2	1,10	1,05–1,20	1,38	1,05–1,88
**1951 plume**						
	^**137**^**Cs** (n = 16)		^**239+240**^**Pu** (n = 6)		^**90**^**Sr** (n = 12)	
	**AM**	**min-max**	**AM**	**min-max**	**AM**	**min-max**
***K***_***n*,*0–20***_	1,07	1,01–1,15	1,07	1,03–1,13	1,78	1,08–2,67
***K***_***n*,*0–30***_	1,08	1,02–1,17	1,07	1,03–1,13	1,83	1,09–2,75

The analysis of coefficient values listed in Table 3 shows that the spread in values for ^137^Cs, ^241^Am and ^239+240^Pu is insignificant for all areas, whereas for ^90^Sr, in fallout plumes, it may reach up to 2 times, and the difference in conventionally ‘background’ areas reaches 1 order of magnitude. Thus, findings allow estimates of a possible content of such radionuclides as ^137^Cs, and ^239+240^Pu in the 0–20 and 0–30 cm soil layers from the data on activities of these radionuclides for the 0–5 cm soil layer with an adequately high reliability by mean values or by maxima if a conservative approach is taken. The estimate of ^90^Sr in this way is not recommended.

## Conclusions

Thus, research into the vertical distribution of radionuclides in soil cover of STS areas located beyond testing site, that is in areas with no nuclear weapons or nuclear effects of radiological warfare agents tested, allowed definition of the distribution nature of radionuclides in soil of these areas. Research has shown that maxima of activity concentrations of radionuclides all over the study area were observed in the 0–5 cm spoil layer rates of the vertical migration of nuclides downward soils, based upon the activity detected, were as follows: ^90^Sr– ^137^Cs– ^239+240^Pu– ^241^Am (the descending series). Activity ratios established for radionuclides in the soil cover layer down to 20 cm and 30 cm to those of radionuclide in the 0–5 cm topsoil allow assessments of the possible content of ^137^Cs and ^239+240^Pu in the 0–20 and 0–30 cm soil layers from the data on activity concentrations of these radionuclides in the 0–5 cm soil layer with a sufficiently high reliability. This is important to develop recommendations for carrying out agricultural activities within STS and a rehabilitation strategy for individual test site areas.
